# From “Aisle” to “Labile”: A Hierarchical National Adult Reading Test Scale Revealed by Mokken Scaling

**DOI:** 10.1037/pas0000091

**Published:** 2015-08-10

**Authors:** Sarah McGrory, Elizabeth J. Austin, Susan D. Shenkin, John M. Starr, Ian J. Deary

**Affiliations:** 1 Alzheimer Scotland Dementia Research Centre and Department of Psychology, University of Edinburgh; 2 Department of Psychology, University of Edinburgh; 3 Geriatric Medicine and Centre for Cognitive Ageing and Cognitive Epidemiology, University of Edinburgh; 4 Alzheimer Scotland Dementia Research Centre, Geriatric Medicine, and Centre for Cognitive Ageing and Cognitive Epidemiology, University of Edinburgh; 5 Geriatric Medicine and Centre for Cognitive Ageing and Cognitive Epidemiology, University of Edinburgh

**Keywords:** Mokken scaling, hierarchical scales, item response theory, premorbid cognitive ability, NART

## Abstract

Decline in cognitive ability is a core diagnostic criterion for dementia. Knowing the extent of decline requires a baseline score from which change can be reckoned. In the absence of prior cognitive ability scores, vocabulary-based cognitive tests are used to estimate premorbid cognitive ability. It is important that such tests are short yet informative, to maximize information and practicability. The National Adult Reading Test (NART) is commonly used to estimate premorbid intelligence. People are asked to pronounce 50 words ranging from easy to difficult but whether its words conform to a hierarchy is unknown. Five hundred eighty-seven healthy community-dwelling older people with known age 11 IQ scores completed the NART as part of the Lothian Birth Cohort 1936 study. Mokken analysis was used to explore item responses for unidimensional, ordinal, and hierarchical scales. A strong hierarchical scale (“mini-NART”) of 23 of the 50 items was identified. These items are invariantly ordered across all ability levels. The validity of the interpretation of this briefer scale’s score as an estimate of premorbid ability was examined using the actual age 11 IQ score. The mini-NART accounted for a similar amount of the variance in age 11 IQ as the full NART (NART = 46.5%, mini-NART = 44.8%). The mini-NART is proposed as a useful short clinical tool to estimate prior cognitive ability. The mini-NART has clinical relevance, comprising highly discriminatory, invariantly ordered items allowing for sensitive measurement, and adaptive testing, reducing test administration time, and patient stress.

Determining the degree of cognitive decline caused by dementia or a normal aging process relies on establishing a valid estimate of prior ability level (Crawford, 1992). There are substantial individual differences in cognitive ability; therefore, it is important to take a person’s prior/premorbid cognitive ability level into account to establish whether there has been a decline. Preferably, this would involve a comparison of current cognitive ability with an actual measure of prior cognitive ability. However, actual premorbid measures of ability are seldom available in clinical situations. This results in the dependence upon estimates of premorbid cognitive function.

A commonly used test for estimating peak premorbid cognitive ability is the National Adult Reading Test (NART) (Nelson, 1982; Nelson & Willison, 1991). This test examines pronunciation of 50 irregular English words of graded difficulty which violate the typical grapheme-phoneme and stress rules (e.g., gauche, thyme), that is, guessing will not provide the correct pronunciation. The shortness of the words ensures that minimal demands are placed on the patient’s current mental capacity (Nelson & O’Connell, 1978). Therefore, successful word reading is thought to depend on premorbid ability and not on current cognitive ability. The NART has been validated as an estimator of premorbid mental ability in mild to moderate dementia (Bright, Jaldow, & Kopelman, 2002; Crawford, Parker, & Besson, 1988; McGurn et al., 2004; Sharpe & O’Carroll, 1991;) and also in normal cognitive aging (Dykiert & Deary, 2013). After controlling for age 11 IQ, mean NART scores do not differ between those with and without mild-to-moderate dementia (McGurn et al., 2004).

The NART comprises words of graded difficulty starting with more commonly used words, such as “ache” and “chord” and becoming more difficult as it progresses to less frequently used words, such as “syncope” and “campanile.” Whereas NART items may be considered as forming an informal hierarchy, as planned by the test’s constructors, it is important to investigate item properties explicitly to determine whether the items conform to a formal hierarchy of difficulty and whether this hierarchy is the same for all respondents (i.e., is the ordering for people with higher levels of ability the same as for those with lower ability levels). The effect of ability level on item ordering was investigated by Deary, Watson, Booth, and Gale (2013) who determined that the strength of hierarchies of item ordering of the Warwick-Edinburgh Mental Well-being Scale varied according to the cognitive ability of the sample. Item *difficulty* represents the ease at which an item is responded to correctly, with mean item scores used to indicate an item’s *difficulty* level. Establishing whether a scale has hierarchical properties adds another dimension to its use other than just using total summed scores. If a scale has hierarchical item ordering it implies that the items are ordered relative to each other and that all are ordered along the latent trait being measured. A hierarchy with the same ordering of *difficulty* for all subgroups from the population of interest, referred to as invariant item ordering (IIO; Sijtsma & Junker, 1996). IIO simplifies the interpretation of the results by avoiding different item ordering between different relevant subgroups which would warrant further analysis to find the reason for these differences (Sijtsma & Hemker, 1998).

From a clinical perspective, hierarchical tests are attractive for their ease of use and scoring (Kempen, Myers, & Powell, 1995). Confirming hierarchies of item *difficulty* has meaningful clinical implications; continuing to test patients on words that they are predictably going to be unable to pronounce correctly may cause undue distress without adding any valuable information. Also, responses to individual items and not just total scores can provide insight into a respondent’s level of ability based on the item’s location in the hierarchy (Watson, Deary, & Austin, 2007). Hierarchical tests have proven valuable in the assessment of several constructs, for example, psychological distress (Watson, Deary, & Shipley, 2008), feeding difficulty in dementia (Watson, 1996), and activities of daily living (Fieo, Watson, Deary, & Starr, 2010; Kempen & Suurmeijer, 1990).

An analogy of climbing a staircase can be used to illustrate the properties of a hierarchical scale. Each step represents an item in a scale. Any given height within the staircase represents the level of latent trait. It follows that you cannot reach the ninth step without having previously climbed the eight steps below; and by having climbed to the ninth step you will not have reached any step above this level.

The degree to which items in a test form a hierarchy can be determined using Mokken scaling analysis which searches multivariate data for unidimensional, ordinal, and hierarchical scales. Mokken scaling is a nonparametric application of item response theory (IRT) that explores the relationship between items and the latent trait (Watson et al., 2012). Mokken scaling analysis can be applied to examine clinically valuable properties of items within scales, including item *discrimination*. *Discrimination* reflects the degree of accuracy with which an item can distinguish between respondents of different levels of the latent trait and indicates the strength of the relationship between an item and the other items within a scale. Considering item *discrimination* allows for the creation of scales with greater precision without having to increase the number of items. For example, Sabourin, Valois, and Lussier (2005) used IRT methods to create a four-item abbreviated form of the Dyadic Adjustment Scale, which was as effective as the original 32 item scale. Similarly, a 10-item scale was derived from the 19-item Feelings Scale without the loss of measurement precision (Edelen & Reeve, 2007).

IRT methods have been applied to two measures of premorbid intelligence: a French language version of the NART, the *f*NART (Mackinnon, Ritchie, & Mulligan, 1999) and the Adult Reading Test (ART; Letz et al., 2003). Mackinnon et al. (1999) used a two-parameter logistic IRT model to examine the measurement properties of the 40-item *f*NART. The *discrimination* of the scale items varied considerably with several of the items contributing little to the assessment of premorbid intelligence. A refined 33-item *f*NART was revealed with the elimination of seven items with poor *discriminatory* power.

Letz et al. (2003) fit a one-parameter logistic (Rasch) model to the items of the ART, adapted from the North American Adult Reading Test (NAART; Blair & Spreen, 1989). Rasch analysis provided an improved ordering of *difficulty* from the original subjective ranking, finding “two” to be one of the least *difficult* items and “demesne” to be the most *difficult* item. Results from this Rasch analysis formed the basis for the implementation of a computerized-adaptive ART whereby items are matched to respondents by *difficulty*. This prevents individuals being presented with items far beyond their ability level helping to reduce frustration or anxiety and minimizing the boredom or carelessness of those with higher ability when faced with very easy items.

The possibility of deriving a briefer scale from the NART from which to estimate premorbid IQ is not new. Beardsall and Brayne (1990) explored the idea of creating a shortened version of the NART. A regression equation was developed based on scores from the first 25 words of the NART to predict scores on the remaining 25 words (i.e., items 26 to 50). This method provided a reasonably accurate estimation of the full NART score with predicted NART and true NART scores correlating strongly, *r* = .93, *p* < .001. While the application of the Short NART left a proportion (23–31%) of the variance unaccounted for, the accuracy with which the Short NART predicted Wechsler Adult Intelligence Scale IQ was effectively equal to that of the full NART (Crawford, Parker, Allan, Jack, & Morrison, 1991). The authors suggest the application of the Short NART with reasonable confidence where helpful or convenient in place of the full scale.

While these studies have analyzed and refined the assessment of premorbid cognitive ability, to our knowledge, there has been no application of Mokken scale analysis to the NART. Therefore the aim of the present study was to examine the item properties and the hierarchical structure of the NART by assessing the fit of the items to Mokken’s monotone homogeneity model (MHM) and the nonintersection of item response functions (IRFs). The IRF is the building block of IRT and represents the probability of endorsing as item as a function of the underlying trait (Fraley, Waller, & Brennan, 2000). When the assumptions of the MHM hold, the IRFs increase as levels of the latent trait increase, meaning that respondents can be ordered with respect to their latent trait level based on the summed total score of their responses. Nonintersection, now more commonly referred to as IIO, is an item ordering property whereby the IRFs for total scores on a set of items do not intersect and is crucial for establishing hierarchical scales. In the case of dichotomous items where IRFs are nonintersecting, IIO (formally known as double monotonicity) can be said to hold. Establishing the fit of the data to these models would allow the use of total scale scores (in the case of the MHM) and individual items (IIO) to assess estimated levels of premorbid cognitive ability. Additionally this analysis aims to determine the contribution of each item. Redundant items can be removed to form a new brief scale.

## Method

### Participants

The Lothian Birth Cohort 1936 (LBC1936) comprises 1091 community-dwelling older adults most of whom completed the Moray House Test No. Twelve (MHT) (Scottish Council for Research in Education (SCRE), 1933) of verbal reasoning at a mean age of 11 as part of the Scottish Mental Survey of 1947 (Scottish Council for Research in Education, 1949; Deary, Whalley, & Starr, 2009). The Scottish Mental Survey of 1947 (SMS1947) measured the mental ability of almost all Scottish schoolchildren born in 1936 and attending school at age 11 years on June 4th 1947 using the MHT. The MHT is a well-validated measure of general intelligence comprising mostly verbal reasoning items with a maximum possible score of 76. Childhood MHT scores were highly correlated with the Stanford-Binet intelligence test, *r* = .81 in boys (*N* = 500) and *r* = .78 in girls (*N* = 500; SCRE, 1933). Between 2004 and 2007 those residing in Edinburgh and the Lothians who may have taken part in the SMS 1947, who were then approximately age 70, were contacted and invited to participate in the LBC1936. The Community Health Index was used to identify potential participants born in 1936. All participants spoke English as their first language.

Social class was derived from the participants’ reported highest occupational level as well as that of participants’ fathers. Social class for the participants was calculated using the Office of Population Censuses and Surveys; Classification of Occupations, 1980. Social class of participants’ fathers was calculated using the General Register Office’s Census, 1951 Classification of Occupations. Both were classified as one of six categories from I (professional) to V (unskilled) with lower numbers designating higher social class. Married women also reported the occupation of their spouses which was used if higher. The recruitment and testing of this cohort has been described in detail elsewhere (Deary, Gow, Pattie, & Starr, 2012; Deary et al., 2007).

Participants in the LBC1936 returned for detailed cognitive and physical testing from age 70 (wave 1, *N* = 1091), and item level responses to the NART were recorded at wave 3 (2012), at a mean age of about 76 years. Age 70 IQ was measured by the MHT (*M* = 65.7, *SD* = 7.7) corrected for age in days at time of testing, and converted to an IQ score (mean IQ = 102.42, *SD* = 13.16). Self-reported medical background was obtained for all participants at the cognitive and physical assessment. After excluding those who had a self-reported clinical history of dementia (*N* = 8) data from all other participants returning at wave 3 with complete NART item level data were included for analysis (*N* = 587, 51% male). Mini Mental State Examination (MMSE) (Folstein, Folstein, & McHugh, 1975) scores indicated that 99.6% of this sample scored ≥23. The characteristics of study participants are shown in Table 1.[Table-anchor tbl1]

### Measures

The 50 items of the NART are scored dichotomously; respondents are either able or unable to pronounce the word correctly. Higher scores (fewer errors) indicate higher premorbid cognitive ability. The NART has high internal consistency (0.90; Crawford et al., 1988), high test-retest reliability (0.98; Crawford, Parker, Stewart, Besson, & Lacey, 1989) and good interrater reliability (0.88; O’Carroll, 1987).

The percentage of respondents correctly pronouncing the NART items was used to indicate level of item *difficulty* with lower percentages indicating greater degree of *difficulty*.

### Mokken Scaling

Mokken scaling analysis was applied to investigate whether the ordering of items by *difficulty* is the same for all respondents, making it invariantly ordered.

The fit of the items to Mokken scaling properties was assessed by examining whether they conformed to the four assumptions; unidimensionality, local stochastic independence, monotonicity, and nonintersection. Mokken scaling analysis was performed using the Mokken package in R (van der Ark, 2007). These assumptions were investigated using a hierarchical clustering algorithm, scalability coefficients, latent monotonicity, and the *H*^*T*^ coefficient.

#### Automated item selection procedure (AISP)

The assessment of unidimensionality involves an automated item selection procedure (AISP) which partitions items into scales, or groups of related items measuring a common latent trait, using a hierarchical clustering algorithm. The AISP is a bottom-up sequential item selection method based on interitem covariances and the strength of the association between the items and the latent trait. The process begins with the selection the pair of items with the highest positive item-pair scalability coefficient (*H*_*ij*_). This selection procedure proceeds until no additional items meet this criterion. From any items remaining unselected a new scale can be formed in the same way. Any items remaining out with a scale are deemed unscalable (Sijtsma & Molenaar, 2002).

#### Scalability coefficients

Item, item-pair, and scale scalability coefficients are computed and used as criteria for partitioning items into scales and as measures of strength of the scales. Item scalability coefficients (*H*_*i*_) express item *discrimination*. This coefficient is equivalent to item-test correlation or a factor loading. Item-pair scalability coefficients (*H*_*ij*_) reflect the joint scalability of item pairs. Scale scalability coefficient (*H*) expresses the strength of the overall scale. A general rule of thumb for interpretation of *H* exists: scales with values below 0.3 are not considered unidimensional, between 0.3 and 0.4 are considered as weak, values between 0.4 and 0.5 are indicative of a medium strength, and values greater than 0.5 can be considered as strong (Mokken, 1971).

#### Latent monotonicity

The assessment of monotonicity is important as it enables the respondents to be ordered on the latent trait with respect to the summed score of the scale. Items violating this assumption can be detected and removed if necessary. To avoid the model being rejected due to trivially small violations only violations greater than the default minimum of 0.03 are considered relevant (van der Ark, 2007).

#### Invariant item ordering

The method to investigate IIO used here is advocated by van der Ark (2012) and can be performed using the Mokken package in R by running the *check.iio* command. Here, all violations of IIO are detected and removed. The item with the largest violation is removed first and the remaining items checked again for IIO violations. This is done iteratively as the exclusion of one item may affect the IIO violations of the remaining items. Scalability coefficient *H*^*T*^ is computed and is considered a measure of the accuracy of item ordering within a scale with a similar rule of thumb for interpretation as *H* (Ligtvoet et al., 2010).

Reliability can be estimated using the Molenaar Sijtsma statistic (MS) (Molenaar & Sijtsma, 1984). MS provides a direct estimate of the test score reliability with MS >0.7 indicative of a reliable scale.

### Graphical Analysis

The R package KernSmoothIRT (Mazza, Punzo, & McGuire, 2014) was used to graphically present item properties. The package applies kernel smoothing in the estimation of item response functions and related graphical analysis. It provides several plotting and analytical methods to consider properties of the items, subjects, and test as a whole. The exploratory nature of the package makes it ideal to be used alongside Mokken analysis since it provides plots which can be helpful when examining the monotonicity and *discrimination* of items. For more details on this package see Mazza et al. (2014).

### Validation

The present study had access to childhood IQ scores which enabled the retrospective validity of the NART items as proxies for prior cognitive ability across the life span to be assessed. The correlation between NART items and prior and concurrent cognitive ability, both measured by converting MHT scores at age 11 and age 70 into IQ scores, was investigated. Regression and correlation analyses were performed using SPSS v. 19.0.

## Results

Table 1 shows descriptive statistics for the sample variables.

Mean (*SD*) total NART score for this sample was 35.3 (7.7), equivalent to an IQ of 112.3 (based on regression equations calculated by Nelson and Willison (1991)). The mean (*SD*) MHT score at age 11 for this sample of the LBC 1936 cohort was 50.6 (11.6) compared with a mean of 36.7 (16.1) for Scotland (*N* = 70,805) (Deary, Gow, Pattie, & Starr, 2012; SCRE, 1949). Converted to an IQ score, the mean IQ for this sample, 0.864 standard deviations above a mean of 100 (*SD* = 15) is 113.

Items ordered from least to most *difficult* in Table 2 demonstrates several inconsistencies between this ordering by mean scores and the test order in this sample. For example, “capon” and “drachm” which are seventh and 33rd in the test administration order, respectively, are the 22nd and 50th item in the ordering by sample mean scores.[Table-anchor tbl2]

The Mokken automated item selection procedure partitioned 38 of the 50 items into one scale, three items into a second scale, and determined the remaining nine items to be unscalable (see Appendix A for a table of items in each scale). The scalability coefficients of the 38 items of scale 1 were examined. All item-pair scalability coefficients (*H*_*ij*_s) were non-negative and all item scalability coefficients were above 0.3, indicating that these 38 items belong in the same unidimensional Mokken scale. There were no significant violations of monotonicity. All 38 items of this abbreviated NART form a Mokken scale meeting MHM criteria (*H* = 0.471, *SE* = 0.017) (see Appendix B for a table of 38 abbreviated NART items ordered by *discrimination*).

These 38 items were examined for violations of nonintersection. Fifteen items violated IIO (hiatus, placebo, procreate, capon, façade, superfluous, deny, simile, banal, assignate, equivocal, puerperal, subtle, gouge, syncope) and were removed.

### The Mini-NART

Removing the items that violated IIO resulted in a 23 item scale (the “mini-NART”) which had no more significant violations of IIO (Table 3). The total scale scalability coefficient for this subset was 0.534 (*SE* = 0.017), indicating a strong Mokken scale. *H*^*T*^ was 0.71, which indicates sufficient accuracy of item ordering within this scale (Ligtvoet, van der Ark, Te Marvelde, & Sijtsma, 2010). Reliability was very high (MS = 0.89).[Table-anchor tbl3]

**Figure 1 fig1:**
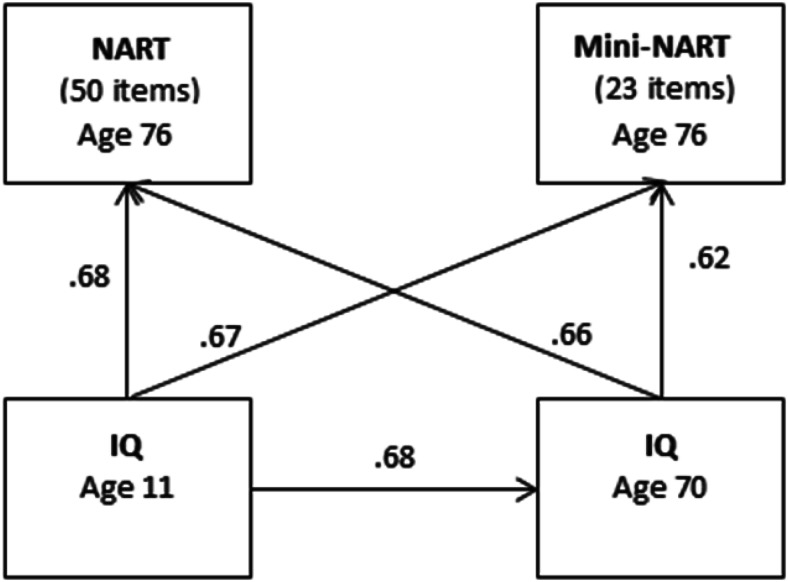
Correlations between age 11 IQ and the NART, Mini-NART, and age 70 IQ. IQ at both ages was assessed using the Moray House Test No. 12. NART = National Adult Reading Test. Mini-NART = Mini National Adult Reading Test.

The pattern of correlations between the NART and the mini-NART and IQ measured at age 11 and age 70 are presented in Figure 1. The NART and the empirically derived mini-NART positively correlated with age 11 IQ (NART: *r* = .68, *P* = <0.001; Mini-NART: *r* = .67, *P* = <0.001). Both original and short versions of the NART correlated with age 70 IQ (NART: *r* = .66, *p* <0.001; mini-NART: *r* = .62, *P* = <0.001).[Fig-anchor fig1]

To investigate the predictive accuracy of the total score from the 23 item mini-NART, regression analyses were carried out. The mini-NART accounted for 44.8% of the explained variability in age 11 IQ-tested 65 years previously in this sample whereas the full version of the NART accounted for 46.5% of the variance. The 38-item abbreviated NART, conforming to the properties of the MHM, accounted for 48.3% of the variance. The regression equations (with 95% confidence interval (CI)) estimating an individual’s premorbid cognitive ability from performance on the mini-NART and NART are presented below:

Mini-NART (23 item IIO scale):
Predicted age 11 IQ = 64.94 (2.345 × Mini-NART score), 95% CI [2.13 × Mini-NART score, 2.56 × Mini-NART score].
For example, for mini-NART score of 20, predicted age 11 IQ = 64.94 + (2.345 × 20) = 111.84, 95% CI [107.54, 116.14].
NART (original 50 item scale):
Predicted age 11 IQ = 55.97+ (1.306 × NART score), 95% CI [1.19 × NART score, 1.42 × NART score].
For example, for NART score of 45, predicted age 11 IQ 55.97 + (1.306 × 45) = 114.74, 95% CI [109.52, 119.87].
For ease of use the table in Appendix C converts NART, abbreviated NART, and mini-NART scores to predicted IQ scores using these regression equations.

### Item Discrimination

**Figure 2 fig2:**
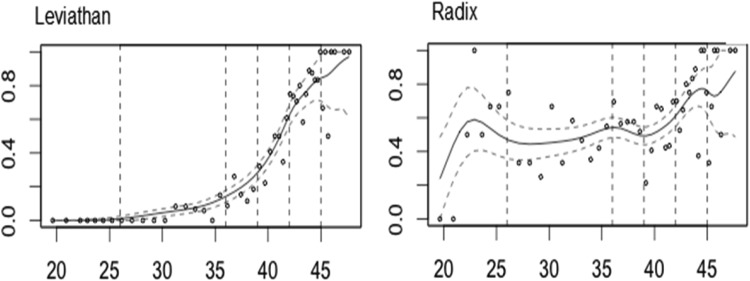
Item response functions illustrating discriminatory power for two NART items: Item 43: “leviathan,” and Item 19: “radix.” The *x*-axis represents the expected scale score. The *y*-axis represents the expected item score. Red dashed lines show the 95% confidence intervals. NART = National Adult Reading Test.

Looking at some items rejected by Mokken scaling it is clear that some NART items are not adequately distinguishing between respondents and are not contributing much to the accurate estimation of premorbid functioning. Figure 2 graphically presents the *discriminatory* power of two items of the NART: “leviathan” (Mini-NART) and “radix” (unscalable). These IRFs, produced by KernSmooth provide a representation of item *discrimination*. The slope here reflects the rate of change, designating the level of effectiveness at any point along the latent trait (DeJong & Molenaar, 1987). The poor *discrimination* value (*H*_*i*_ = 0.001) of item 19 (“radix”) is reflected in the relatively flat IRF. This means that large differences in ability are associated with very modest changes in the probability of correctly pronouncing with “radix.” Practically speaking, two people of different levels of ability are likely to achieve the same score on this item. This item adds little information to the overall estimate of premorbid cognitive ability as some respondents of different levels of ability have similar response profiles. The curve of item 43 (“leviathan”) is very steep in the region of higher ability with small differences in ability at this level associated with substantial differences in the likelihood of correctly pronouncing the word.[Fig-anchor fig2]

## Discussion

The present study investigated the hierarchical nature of the NART by determining whether the data conformed to the assumptions of the MHM and IIO in 587 mostly healthy older adults with prior IQ measured at age 11. It demonstrated the utility of Mokken scaling and graphical analyses in exploring item level responses in the NART.

Two subscales within the NART were revealed: (a) a 38 item abbreviated NART scale conforming to the MHM, and (b) a 23 item mini-NART with IIO. The items in the abbreviated NART can be stochastically ordered by degree of latent trait. However this ordering is not invariant across respondents of different levels of latent trait, that is, the total score of this abbreviated NART, but not individual items, can be used by clinicians and researchers to obtain an estimation of a respondent’s level of premorbid cognitive ability.

The mini-NART, comprising only items strongly related to the latent trait with good *discrimination* values, conforms to a strong and invariantly ordered hierarchy. This adds value and clinical relevance to a scale since it implies a consistent ordering of items which is invariant for all values of the latent trait. Individual items within the mini-NART can be used to approximate a respondent’s level of premorbid cognitive ability. A person’s estimated prior cognitive ability can be represented by the score on a single item in the Mini-NART, the most *difficult* item correctly responded to. This scale could be applied adaptively whereby only a section of the NART either in the higher or lower *difficulty* range of the scale needs to be applied, according to the ability of the individual patient. The test can be administered in order of ascending *difficulty* starting with “aisle” or descending *difficulty* starting with “labile.” For example, a participant who is able to correctly pronounce “labile” or “sidereal” would most likely be able to pronounce all other (less *difficult*) items in the scale. Likewise, any participant unable to correctly pronounce “aisle” or “debt” would most likely be unable to correctly pronounce any of the other (more *difficult*) words.

Administering IIO scales adaptively can help to reduce the time needed to test patients, reducing the burden placed on the patient and helping to diminish the stress or frustration of the patient (van der Lee, Roorda, Beckerman, Lankhorst, & Bouter, 2002). Although the NART in full is a relatively quick scale to administer the reading of progressively more difficult and infrequently encountered words aloud may still cause embarrassment and anxiety among those who are experiencing difficulty. Participants with early dementia or mild cognitive impairment with awareness of declining cognitive abilities are likely to be anxious facing a lengthy test battery. Shorter tests with less potential for distress and embarrassment may reduce the likelihood of participants withdrawing from testing, and may be particularly useful in clinical (medical) environments where time is limited. Adaptive testing or tailored assessment appears to be increasingly appealing in addressing the need for quick and reliable measurement. Ware et al. (2003) reported that the use of an adaptive form of the Headache Impact Survey performed better that the traditional version in terms of reducing respondent burden, measuring change over time and in test reliability and validity. Like the Rasch-derived computerized-adaptive ART (Letz et al., 2003), the mini-NART can be applied adaptively but, importantly, without the expense and practical implications of testing patients with a computerized test.

IRT methods can be used to ensure a scale is measuring what it is designed to measure (Langenbucher et al., 2004; Noerholm et al., 2004). With regard to the NART, 12 items were identified that did not conform to the unidimensional MHM, indicating that in this sample the NART in full includes items not measuring the same latent trait. Also, Mokken scaling suggests that “drachm,” “topiary,” and ‘prelate” form a separate cluster which may measure something other than premorbid cognitive ability. The inclusion of these items may mean that the total NART score does not solely reflect premorbid cognitive ability. Rasch analysis of the ART, which has several items in common with the NART, identified “aeon” and “banal” as candidates for removal from mis-fit statistics (Letz et al., 2003). Neither of these items was retained in the mini-NART which adds validity to the removal of these items from the full NART.

By removing poor *discriminatory* items, the mini-NART with similar predictive accuracy was identified. We have found that adding extra items to the mini-NART does not increase the amount of variance of age 11 IQ explained in this sample. This mini-NART, like the Short NART, offers predictive accuracy effectively equal to that of the full scale. However the mini-NART avoids the complications of the Short NART testing process. Beardsall and Brayne (1990) suggest testing patients on the first half (Short NART) and applying a regression equation to predict the full score for patients scoring between 12 and 20 on this Short NART. If a patient scores less than 12 on the Short NART this score should be taken as the full NART score and for those scoring over 20 the full NART should be administered to determine their score. To observe these discontinuation rules a tally of errors must be kept during testing. Short NART total scores must then be converted to a NART error score before premorbid ability can be estimated. The mini-NART requires no extra calculations and has the distinct advantage of being a hierarchical scale.

One limitation of the mini-NART as a means of estimating premorbid cognitive ability is that with only 23 words, it is not as finely graded as the full 50 item scale or the 38 item abbreviated NART. With only 23 items it may not differentiate as efficiently between the higher levels of cognitive ability since its ceiling level of 23 items is predictive of an IQ score of 119. In this sample of 587 Participants 59 have IQ scores greater than 119. However, using the full 50- item NART, this ceiling is only extended by approximately two IQ points to 121. An estimated IQ based on a maximum score should be interpreted as a lower-limit estimate only with a mini-NART score of 23, indicative of an IQ of 119 or higher.

The present analysis demonstrates the utility of IRT in examining item properties of established scales and how this insight can be used in the development of a shorter hierarchical scale. This study applied novel methods in a well-characterized sample with relatively large numbers. A particular strength of this study is the availability of a valid intelligence test score from age 11 for the sample, which ensures the scores are free from age-related decline. This permitted the validity of the mini-NART to be assessed using the actual premorbid cognitive ability. Dykiert and Deary (2013) and Crawford, Deary, Starr, and Whalley (2001) also utilized the prior ability of the LBC to examine the retrospective validity of the NART. Due to the rarity of actual premorbid ability data previous validation studies typically compared NART performance with measures of current abilities (Crawford et al., 1989; Nelson, 1982).

Some limitations of the study should be noted. The self-selected LBC1936 cohort is not fully representative of the population. First, the cohort is geographically restricted. The LBC 1936 cohort is also somewhat restricted in range with regards to childhood cognitive ability. The individuals in this sample are of a higher than average ability level, scoring almost 14 MHT points higher at age 11 than their peers across Scotland (Scottish Council for Research in Education, 1949; Deary, Gow, Pattie, & Starr, 2012). This is reflected in how few items there are with low percent correct in the NART in this above-average ability sample. Performing the same analysis on a more representative sample with lower cognitive abilities with fewer participants approaching ceiling performance for many items would be a valuable extension to this analysis. Also, this analysis was carried out using a sample of elderly participants without self-reported dementia. The self-reported history of dementia is subject to the accuracy of recall. However with only 1% of participants scoring less than 24 points on the MMSE, suggesting possible dementia, the sample is mostly cognitively healthy. To examine the generalizability of these findings it is necessary to examine the accuracy of the mini-NART in a cross-validation sample before applying the scale in clinical practice. Replication using participants with a range of abilities, and diagnoses of dementia and mild cognitive impairment is necessary to investigate the performance of the mini-NART in pathological cognitive decline. Also, the NART and mini-NART account for less than 50% of the reliable variance in premorbid cognitive ability leaving a significant percentage unaccounted for. However, this is a lower-bound estimate which does not account for restriction of range or measurement error.

The value of *H*^*T*^ here is very high and, as such, it is worth noting that in some cases elevated *H*^*T*^ values can be caused by violations of local stochastic independence (Watson, Wang, & Thompson, 2014). Local stochastic independence is violated when items within a scale are linked (i.e., the response to one item is dependent on the response to another). In the case of the NART local stochastic independence is very unlikely to have been violated since the responses are not dependent on each other.

One possible reason to explain why IIO did not hold for some items may reflect how people’s knowledge of some of the more *difficult* and unusual words, some of which depend on specialist experience (e.g., medical terms like syncope, puerperal), is quite unpredictable, which will have an effect on responses. This could also help to explain the inconsistencies between the item ordering by mean scores and the test administration order. The effect of regional variation in pronunciation is also likely to contribute this irregular response ordering. With regard to unscalable items, it is possible that agreement between raters could be partly responsible. Crawford et al. (1989) found “aeon” to have an agreement rate closer to chance than perfect agreement, which could help to explain why this item did not follow the typical pattern of response one would expect.

## Conclusions

Good scales with good psychometric properties, including IIO, are sought for accurate assessment in clinical practice and this paper demonstrates how Mokken scaling can help contribute to this goal. Mokken scaling analysis revealed that some NART items do not contribute to the measurement of premorbid cognitive ability in this sample and identified other items whose contribution is low. This analysis identified a useful, unidimensional, and highly *discriminatory* scale within the NART; the mini-NART, a hierarchical subset of 23 invariantly ordered items. While further research to support the validity of the mini-NART, particularly in populations more representative of the general population, is necessary, the 23-item scale is presented as a promising alternative to the original NART for both clinicians and researchers. The mini-NART could prove to be of clinical and practical benefit in the estimation of premorbid cognitive ability.

## Figures and Tables

**Table 1 tbl1:** Baseline Sample Characteristics

	Mean	*SD*
Age	76.3	0.7
Sex		
Male (%)	51.1	
Female (%)	48.9	
Age 11 IQ	101.5	14.9
Age 70 IQ	102.4	13.2
Age 11 MHT	50.6	11.6
Age 70 MHT	65.7	7.7
MMSE	28.7	1.5
NART	35.3	7.7
Father’s SES	2.9	0.9
Participant’s SES	2.5	0.9
Education (years)	10.8	1.2
*Note.* *SD* = standard deviation; MMSE = Mini-Mental State Examination; NART = National Adult Reading Test. IQ calculated from MHT (Moray House Test) score corrected for age in days at time of testing and converted to IQ scale. Father’s SES (socio-economic status) is participants’ father’s social class when the participant was 11 years old.		

**Table 2 tbl2:** NART Items Ordered by Percentage of Correct Responses in LBC1936 (n = 587) (From Least to Most Difficult)

NART order	Item	Correct (%)	NART order	Item	Correct (%)
2	ACHE	99.3	32	ZEALOT	80.6
4	AISLE	99.1	28	BANAL	79.4
10	DEBT	99.0	15	CATACOMB	78.4
1	CHORD	99.0	16	GAOLED	76.8
6	PSALM	98.5	31	FACADE	75.1
18	HEIR	98.0	30	CELLIST	72.9
3	DEPOT	97.4	42	TOPIARY	72.6
9	NAUSEA	97.4	29	QUADRUPED	69.5
5	BOUQUET	96.9	36	ABSTEMIOUS	67.6
14	NAIVE	93.0	41	GAUCHE	63.2
23	PROCREATE	93.0	40	AVER	58.4
8	DENY	91.6	37	DETENTE	55.0
25	GOUGE	90.6	38	IDYLL	47.5
35	PLACEBO	89.9	19	RADIX	44.1
20	ASSIGNATE	89.8	34	AEON	42.4
11	COURTEOUS	89.4	39	PUERPERAL	40.7
22	SUBTLE	89.1	44	BEATIFY	37.3
12	RAREFY	88.6	43	LEVIATHAN	35.7
17	THYME	86.7	45	PRELATE	31.7
13	EQUIVOCAL	85.8	48	SYNCOPE	28.8
27	SIMILE	85.7	47	DEMESNE	22.0
7	CAPON	85.3	50	CAMPANILE	17.4
26	SUPERFLUOUS	84.7	46	SIDEREAL	17.2
21	HIATUS	84.7	49	LABILE	14.1
24	GIST	83.1	33	DRACHM	13.8
*Note.* NART = National Adult Reading Test; NART order = item number of word order in current NART testing procedure/hierarchy (i.e. Item 1, “chord,” presented first); Correct (%) = percentage of respondents correctly pronouncing the items with higher percentages indicating lower *difficulty*.					

**Table 3 tbl3:** Item Difficulty and Discrimination of the Mini-NART

NART order	Item	*H*_i_	Correct (%)
4	AISLE	0.570	99.1
10	DEBT	0.592	99.0
6	PSALM	0.409	98.5
18	HEIR	0.508	98.0
3	DEPOT	0.391	97.4
9	NAUSEA	0.483	97.4
5	BOUQUET	0.455	96.9
14	NAIVE	0.502	93.0
17	THYME	0.484	86.7
24	GIST	0.534	83.1
16	GAOLED	0.462	76.8
30	CELLIST	0.526	72.9
29	QUADRUPED	0.519	69.5
36	ABSTEMIOUS	0.541	67.6
41	GAUCHE	0.502	63.2
40	AVER	0.476	58.4
37	DETENTE	0.550	55.0
38	IDYLL	0.523	47.5
44	BEATIFY	0.561	37.3
43	LEVIATHAN	0.622	35.7
47	DEMESNE	0.701	22.0
46	SIDEREAL	0.606	17.2
49	LABILE	0.581	14.1
		*H* = 0.534	
*Note.* NART = National Adult Reading Test; NART order = item number of word order in current NART testing; *H*_*i*_ = item scalability coefficient (item *discrimination*) with higher values indicating greater discrimination; *H* = scale scalability coefficient with higher values indicating greater scalability; Correct (%) = percentage of respondents correctly pronouncing the items with higher percentages indicating lower *difficulty*.			

## A Partitioning of Items by the Automated Item Selection Procedure

**Table:**

Scale 1	Scale 2	Scale 0
DEPOT	DRACHM	CHORD
AISLE	TOPIARY	ACHE
BOUQUET	PRELATE	COURTEOUS
PSALM		RAREFY
CAPON		CATACOMB
DENY		RADIX
NAUSEA		ZEALOT
DEBT		AEON
EQUIVOCAL		CAMPANILE
NAIVE		
GAOLED		
THYME		
HEIR		
ASSIGANTE		
HIATUS		
SUBTLE		
PROCREATE		
GIST		
GOUGE		
SUPERFLUOS		
SIMILE		
BANAL		
QUADRUPED		
CELLIST		
FACADE		
PLACEBO		
ABSTEMIOUS		
DETENTE		
IDYLL		
PUEPERAL		
AVER		
GAUCHE		
LEVIATHAN		
BEATIFY		
SIDEREAL		
DEMESNE		
SYNCOPE		
LABILE		

## B Items of the Abbreviated NART Ordered by Discrimination (*H_i_*)

**Table:**

Item	Label	*H*_*i*_	Item	Label	*H*_*i*_
10	DEBT	0.694	22	SUBTLE	0.496
47	DEMESNE	0.673	41	GAUCHE	0.481
43	LEVIATHAN	0.604	40	AVER	0.453
46	SIDEREAL	0.601	24	GIST	0.436
4	AISLE	0.597	14	NAIVE	0.435
49	LABILE	0.582	5	BOUQUET	0.412
44	BEATIFY	0.558	20	ASSIGNATE	0.406
31	FACADE	0.556	3	DEPOT	0.405
37	DETENTE	0.543	16	GAOLED	0.400
36	ABSTEMIOUS	0.537	23	PROCREATE	0.398
18	HEIR	0.536	25	GOUGE	0.392
38	IDYLL	0.529	35	PLACEBO	0.377
26	SUPERFLOUS	0.524	8	DENY	0.375
9	NAUSEA	0.517	13	EQUIVOCAL	0.374
48	SYNCOPE	0.513	17	THYME	0.365
30	CELLIST	0.502	6	PSALM	0.364
39	PUERPERAL	0.500	28	BANAL	0.334
27	SIMILE	0.500	21	HIATUS	0.318
29	QUADRUPED	0.497	7	CAPON	0.309

## C Conversion of NART, Abbreviated NART and Mini-NART Scores to Predicted Premorbid IQ

**Table:**

NART Score	Predicted premorbid IQ	Abbreviated NART score	Predicted premorbid IQ	Mini-NART score	Predicted premorbid IQ
50	121.27	38	116.26	23	118.88
49	119.96	37	114.73	22	116.53
48	118.66	36	113.20	21	114.19
47	117.35	35	111.67	20	111.84
46	116.05	34	110.14	19	109.50
45	114.74	33	108.61	18	107.15
44	113.43	32	107.07	17	104.81
43	112.13	31	105.54	16	102.46
42	110.82	30	104.01	15	100.12
41	109.52	29	102.48	14	97.77
40	108.21	28	100.95	13	95.43
39	106.90	27	99.42	12	93.08
38	105.60	26	97.89	11	90.74
37	104.29	25	96.36	10	88.39
36	102.90	24	94.83	9	86.05
35	101.68	23	93.30	8	83.70
34	100.37	22	91.76	7	81.36
33	99.07	21	90.23	6	79.01
32	97.76	20	88.70	5	76.67
31	96.46	19	87.17	4	74.32
30	95.15	18	85.64	3	71.98
29	93.84	17	84.11	2	69.63
28	92.54	16	82.58	1	67.29
27	91.23	15	81.15		
26	89.93	14	79.52		
25	88.62	13	77.98		
24	87.31	12	76.45		
23	86.01	11	74.92		
22	84.70	10	73.39		
21	83.40	9	71.86		
20	82.09	8	70.33		
19	80.78	7	68.80		
18	79.48	6	67.27		
17	78.17	5	65.74		
16	76.87	4	64.21		
15	75.56	3	62.68		
14	74.25	2	61.14		
13	72.95	1	59.61		
12	71.64				
11	70.34				
10	69.03				
9	67.72				
8	66.42				
7	65.11				
6	63.81				
5	62.50				
4	61.19				
3	59.89				
2	58.58				
1	57.28				

## References

[c1] Beardsall L., & Brayne C. (1990). Estimation of verbal intelligence in an elderly community: A prediction analysis using a shortened NART. British Journal of Clinical Psychology, 29, 83–90. 10.1111/j.2044-8260.1990.tb00851.x 2310873

[c2] Blair J. R., & Spreen O. (1989). Predicting premorbid IQ: A revision of the National Adult Reading Test. Clinical Neuropsychologist, 3, 129–136. 10.1080/13854048908403285

[c3] Bright P., Jaldow E., & Kopelman M. D. (2002). The National Adult Reading Test as a measure of premorbid intelligence: A comparison with estimates derived from demographic variables. Journal of the International Neuropsychological Society, 8, 847–854. 10.1017/S1355617702860131 12240749

[c4] Crawford J. R. (1992). Current and premorbid intelligence measures in neuropsychological assessment In Crawford J. R., McKinlay W., & Parker D. M. (Eds.), A handbook of neuropsychological assessment (pp. 21–49). London: Erlbaum.

[c5] Crawford J. R., Deary I. J., Starr J., & Whalley L. J. (2001). The NART as an index of prior intellectual functioning: A retrospective validity study covering a 66-year interval. Psychological Medicine, 31, 451–458. 10.1017/S0033291701003634 11305853

[c6] Crawford J. R., Parker D. M., Allan K. M., Jack A. M., & Morrison F. M. (1991). The Short NART: Cross-validation, relationship to IQ and some practical considerations. British Journal of Clinical Psychology, 30, 223–229. 10.1111/j.2044-8260.1991.tb00940.x 1933041

[c7] Crawford J. R., Parker D. M., & Besson J. A. (1988). Estimation of premorbid intelligence in organic conditions. The British Journal of Psychiatry, 153, 178–181. 10.1192/bjp.153.2.178 2978378

[c8] Crawford J. R., Parker D. M., Stewart L. E., Besson J. A. O., & Lacey G. (1989). Prediction of WAIS IQ with the National Adult Reading Test: Cross-validation and extension. British Journal of Clinical Psychology, 28, 267–273. 10.1111/j.2044-8260.1989.tb01376.x

[c9] Deary I. J., Gow A. J., Pattie A., & Starr J. M. (2012). Cohort profile: The Lothian Birth Cohorts of 1921 and 1936. International Journal of Epidemiology, 41, 1576–1584. 10.1093/ije/dyr197 22253310

[c10] Deary I. J., Gow A. J., Taylor M. D., Corley J., Brett C., Wilson V., . . . Starr J. M. (2007). The Lothian Birth Cohort 1936: A study to examine influences on cognitive ageing from age 11 to age 70 and beyond. BMC Geriatrics, 7, 28 10.1186/1471-2318-7-28 18053258 PMC2222601

[c11] Deary I. J., Watson R., Booth T., & Gale C. R. (2013). Does cognitive ability influence responses to the Warwick-Edinburgh Mental Well-Being Scale? Psychological Assessment, 25, 313–318. 10.1037/a0030834 23230851 PMC3744814

[c12] Deary I. J., Whalley L. J., & Starr J. M. (2009). A lifetime of intelligence: Follow-up studies of the Scottish Mental Surveys of 1932 and 1947. Washington, DC: American Psychological Association 10.1037/11857-000

[c13] DeJong A., & Molenaar I. W. (1987). An application of Mokken’s model for stochastic, cumulative scaling in psychiatric research. Journal of Psychiatric Research, 21, 137–149. 10.1016/0022-3956(87)90014-8 3585804

[c14] Dykiert D., & Deary I. J. (2013). Retrospective validation of WTAR and NART scores as estimators of prior cognitive ability using the Lothian Birth Cohort 1936. Psychological Assessment, 25, 1361–1366. 10.1037/a0033623 23815111

[c15] Edelen M. O., & Reeve B. B. (2007). Applying item response theory (IRT) modeling to questionnaire development, evaluation, and refinement. Quality of Life Research: An International Journal of Quality of Life Aspects of Treatment, Care & Rehabilitation, 16, 5–18. 10.1007/s11136-007-9198-0 17375372

[c16] Fieo R., Watson R., Deary I. J., & Starr J. M. (2010). A revised activities of daily living/instrumental activities of daily living instrument increases interpretive power: Theoretical application for functional tasks exercise. Gerontology, 56, 483–490. 10.1159/000271603 20051661

[c17] Folstein M. F., Folstein S. E., & McHugh P. R. (1975). Mini-mental state. Journal of Psychiatric Research, 12, 189–198. 10.1016/0022-3956(75)90026-6 1202204

[c18] Fraley R. C., Waller N. G., & Brennan K. A. (2000). An item response theory analysis of self-report measures of adult attachment. Journal of Personality and Social Psychology, 78, 350–365. 10.1037/0022-3514.78.2.350 10707340

[c50] General Register Office Census 1951 England and Wales (Occupation Tables), London, HMSO, 1956.

[c19] Kempen G. I. J. M., Myers A. M., & Powell L. E. (1995). Hierarchical structure in ADL and IADL: Analytical assumptions and applications for clinicians and researchers. Journal of Clinical Epidemiology, 48, 1299–1305. 10.1016/0895-4356(95)00043-7 7490592

[c20] Kempen G. I. J. M., & Suurmeijer T. P. (1990). The development of a hierarchical polychotomous ADL-IADL scale for noninstitutionalized elders. The Gerontologist, 30, 497–502. 10.1093/geront/30.4.497 2394384

[c21] Langenbucher J. W., Labouvie E., Martin C. S., Sanjuan P. M., Bavly L., Kirisci L., & Chung T. (2004). An application of item response theory analysis to alcohol, cannabis, and cocaine criteria in. DSM–IV. Journal of Abnormal Psychology, 113, 72–80. 10.1037/0021-843X.113.1.72 14992659

[c22] Letz R., DiIorio C. K., Shafer P. O., Yeager K. A., Henry T. R., & Schomer D. L. (2003). A computer-based reading test for use as an index of premorbid general intellectual level in North American English-speaking adults. Neurotoxicology, 24, 503–512. 10.1016/S0161-813X(03)00076-7 12900063

[c23] Ligtvoet R., van der Ark L. A., Te Marvelde J. M., & Sijtsma K. (2010). Investigating an invariant item ordering for polytomously scored items. Educational and Psychological Measurement, 70, 578–595. 10.1177/0013164409355697

[c24] Mackinnon A., Ritchie K., & Mulligan R. (1999). The measurement properties of a French language adaptation of the National Adult Reading Test. International Journal of Methods in Psychiatric Research, 8, 27–38. 10.1002/mpr.54

[c25] Mazza A., Punzo A., & McGuire B. (2014). KernSmoothIRT: An R package for kernel smoothing in item response theory. Journal of Statistical Software, 58, 1–34.

[c26] McGurn B., Starr J. M., Topfer J. A., Pattie A., Whiteman M. C., Lemmon H. A., . . . Deary I. J. (2004). Pronunciation of irregular words is preserved in dementia, validating premorbid IQ estimation. Neurology, 62, 1184–1186. 10.1212/01.WNL.0000103169.80910.8B 15079021

[c27] Mokken R. J. (1971). A theory and procedure of scale analysis: With applications in political research. The Hague. Mouton, Berlin: De Gruyter 10.1515/9783110813203

[c28] Molenaar I. W., & Sijtsma K. (1984). Internal consistency and reliability in Mokken’s nonparametric item response model. Tijdschrift voor Onderwijsresearch, 9, 257–268.

[c29] Nelson H. E. (1982). National Adult Reading Test (NART): Test manual. Windsor: NFER-Nelson.

[c30] Nelson H. E., & O’Connell A. (1978). Dementia: The estimation of premorbid intelligence levels using the New Adult Reading Test. Cortex: A Journal Devoted to the Study of the Nervous System and Behavior, 14, 234–244. 10.1016/S0010-9452(78)80049-5 679704

[c31] Nelson H. E., & Willison J. R. (1991). Restandardisation of the NART against the WAIS–R In Nelson H. E. (Ed.), National Adult Reading Test (NART) test manual, (pp. 13–23). Windsor: NFER-Nelson.

[c32] Noerholm V., Groenvold M., Watt T., Bjorner J. B., Rasmussen N. A., & Bech P. (2004). Quality of life in the Danish general population—Normative data and validity of WHOQOL-BREF using Rasch and item response theory models. Quality of Life Research: An International Journal of Quality of Life Aspects of Treatment, Care & Rehabilitation, 13, 531–540. 10.1023/B:QURE.0000018485.05372.d6 15085925

[c33] O’Carroll R. E. (1987). The inter-rater reliability of the National Adult Reading Test (NART): A pilot study. British Journal of Clinical Psychology, 26, 229–230. 10.1111/j.2044-8260.1987.tb01352.x 3664042

[c51] Office of Population Censuses and Surveys Classification of Occupations, HMSO, London (1980).

[c34] Sabourin S., Valois P., & Lussier Y. (2005). Development and validation of a brief version of the dyadic adjustment scale with a nonparametric item analysis model. Psychological Assessment, 17, 15–27. 10.1037/1040-3590.17.1.15 15769225

[c35] Scottish Council for Research in Education (1933). The intelligence of Scottish children: A national survey of an age-group. London, England: University of London Press.

[c36] Scottish Council for Research in Education (1949). The Trend of Scottish Intelligence. London: University of London Press.

[c37] Sharpe K., & O’Carroll R. (1991). Estimating premorbid intellectual level in dementia using the National Adult Reading Test: A Canadian study. British Journal of Clinical Psychology, 30, 381–384. 10.1111/j.2044-8260.1991.tb00962.x 1777763

[c38] Sijtsma K., & Hemker B. T. (1998). Nonparametric polytomous IRT models for invariant item ordering, with results for parametric models. Psychometrika, 63, 183–200. 10.1007/BF02294774

[c39] Sijtsma K., & Junker B. W. (1996). A survey of theory and methods of invariant item ordering. British Journal of Mathematical and Statistical Psychology, 49, 79–105. 10.1111/j.2044-8317.1996.tb01076.x 8652418

[c40] Sijtsma K., & Molenaar I. W. (2002). Introduction to nonparametric item response theory. Thousand Oaks, CA: Sage.

[c41] van der Ark L. A. (2007). Mokken scale analysis in R. Journal of Statistical Software, 20, 1–19.

[c42] van der Ark L. A. (2012). New developments in Mokken scaling analysis in R. Journal of Statistical Software, 48, 1–27.

[c43] van der Lee J. H., Roorda L. D., Beckerman H., Lankhorst G. J., & Bouter L. M. (2002). Improving the Action Research Arm test: A unidimensional hierarchical scale. Clinical Rehabilitation, 16, 646–653. 10.1191/0269215502cr534oa 12392340

[c44] Ware J. E. Jr., Kosinski M., Bjorner J. B., Bayliss M. S., Batenhorst A., Dahlöf C. G., . . . Dowson A. (2003). Applications of computerized adaptive testing (CAT) to the assessment of headache impact. Quality of Life Research: An International Journal of Quality of Life Aspects of Treatment, Care & Rehabilitation, 12, 935–952. 10.1023/A:1026115230284 14651413

[c45] Watson R. (1996). The Mokken scaling procedure (MSP) applied to the measurement of feeding difficulty in elderly people with dementia. International Journal of Nursing Studies, 33, 385–393. 10.1016/0020-7489(95)00058-5 8836763

[c46] Watson R., Deary I., & Austin E. (2007). Are personality trait items reliably more or less ‘difficult’? Mokken scaling of the NEO-FFI. Personality and Individual Differences, 43, 1460–1469. 10.1016/j.paid.2007.04.023

[c47] Watson R., Deary I. J., & Shipley B. (2008). A hierarchy of distress: Mokken scaling of the GHQ-30. Psychological Medicine, 38, 575–579. 10.1017/S003329170800281X 18226289

[c48] Watson R., van der Ark L. A., Lin L. C., Fieo R., Deary I. J., & Meijer R. R. (2012). Item response theory: How Mokken scaling can be used in clinical practice. Journal of Clinical Nursing, 21, 2736–2746. 10.1111/j.1365-2702.2011.03893.x 21883577

[c49] Watson R., Wang W., & Thompson D. R. (2014). Violations of local stochastic independence exaggerate scalability in Mokken scaling analysis of the Chinese Mandarin SF-36. Health and Quality of Life Outcomes, 12, 149 10.1186/s12955-014-0149-5 25358430 PMC4220047

